# A case of colonic MALT lymphoma with intra-abdominal abscess and lung metastasis: A case report

**DOI:** 10.1097/MD.0000000000035778

**Published:** 2023-10-27

**Authors:** Kangkook Lee, Jin Wook Lee, Hye Ra Jung, Myeongsoon Park, Kwang Bum Cho, Ju Yup Lee

**Affiliations:** a Division of Gastroenterology, Department of Internal Medicine, Keimyung University Dongsan Hospital, Keimyung University School of Medicine, Daegu, Korea; b Department of Pathology, Keimyung University Dongsan Hospital, Keimyung University School of Medicine, Daegu, Korea.

**Keywords:** case report, colon, lymphoma, neoplasm metastasis, non-Hodgkin

## Abstract

**Rationale:**

Colonic mucosa-associated lymphoid tissue (MALT) lymphoma is an unusual subtype comprising only 2.5% of all MALT lymphomas. Most cases of colonic MALT lymphoma are diagnosed at an early stage. Therefore, the clinical features of advanced-stage colonic MALT lymphoma have seldom been reported, and the endoscopic findings are not well established. In this study, we report the clinical and endoscopic characteristics of stage IV colonic MALT lymphoma and highlight the importance of repeat biopsy to figure out this rare disease.

**Patient concerns:**

The patient was a 68-year-old male complaining of hematochezia and lower left quadrant abdominal pain for the past 3 days.

**Diagnoses:**

The patient had 3 masses and friable mucosal lesions in the colon. With the first colonoscopy and biopsy, he was initially diagnosed as having eosinophilic colitis. However, the first treatment with steroids did not show any response. Because of atypical clinical features and colonoscopic findings, a second colonoscopy and a repeat biopsy were performed, and the results were consistent with colonic MALT lymphoma arising in the colon. The patient was finally diagnosed with stage IV colonic MALT lymphoma accompanied by multiple distant metastases.

**Interventions and outcomes:**

The patient started to receive chemotherapy with a combination regimen of cyclophosphamide, vincristine, and prednisolone. The follow-up study after 3 months showed stable disease status based on response evaluation criteria in solid tumors.

**Lessons:**

This case report presents atypical clinical characteristics and colonoscopic findings of stage IV colonic MALT lymphoma. Clinical suspicion and repeat biopsy should be considered to diagnose this rare and diagnostically challenging cancer.

## 1. Introduction

Mucosa-associated lymphoid tissue (MALT) lymphoma, a type of marginal zone lymphoma (MZL), is the third-most common type of B-cell non-Hodgkin lymphoma, accounting for approximately 6% to 8% of all non-Hodgkin lymphomas.^[1]^ Chronic infection and autoimmune conditions are associated with MZLs. *Helicobacter pylori (H. pylori*) is a well-known predisposing factor for gastric MALT lymphoma. However, it is unclear whether other infectious agents have been associated with site-specific extranodal MZLs.^[2]^ The most frequently involved organ in MALT lymphoma is the stomach. Therefore, the clinical features, treatment, and prognosis of gastric MALT lymphoma have been well established.^[3]^ Colonic MALT lymphoma is the least common type of gastrointestinal MALT lymphoma, with little reported clinical data available.^[4]^ Because of its indolent characteristics, most cases of colonic MALT lymphoma are diagnosed at an early stage.^[5]^ Hence, the clinical features of advanced-stage colonic MALT lymphoma have rarely been reported. Here, we present the case of a patient diagnosed with stage IV colonic MALT lymphoma that was initially misdiagnosed as eosinophilic colitis.

## 2. Case presentation

**Figure 1.**
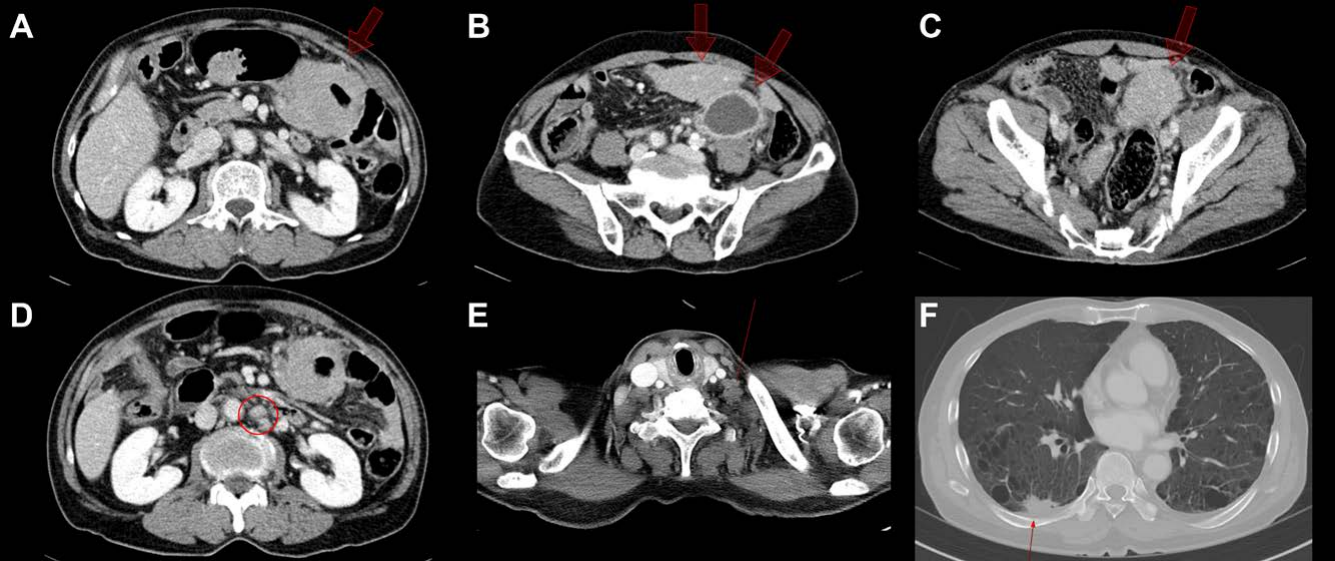
Computed tomography scan showing 3 masses with soft tissue density encasing the transverse and sigmoid colon (white arrows in A–C) and enlarged lymph nodes at the mesentery (white arrow in D) and supraclavicular fossa (blue arrow in E). A pleural mass (white arrow in F) and a 4.5 cm sized intra-abdominal abscess (blue arrow in B) are also seen.

A 68-year-old male patient was admitted to the hospital with complaints of hematochezia and lower left quadrant abdominal pain for the past 3 days. He had a history of cerebral infarction and was taking medications for hypertension and benign prostatic hyperplasia. His blood pressure was 135/85 mm Hg, heart rate was 85 beats/min, respiratory rate was 20 breaths/min, and body temperature was 36.7 °C. The patient’s conjunctiva was pale on physical examination, and the abdomen was soft and non-distended. He had tenderness in the lower left quadrant of the abdomen. The patient’s laboratory tests were as follows: white blood cell count 9400/µL, hemoglobin 10.6 g/dL, platelet count 322 × 10^3^ µL, C-reactive protein 13.37 mg/dL, aspartate aminotransferase 51 IU/L, alanine aminotransferase 62 IU/L, alkaline phosphatase 102 IU/L, total bilirubin 0.42 mg/dL, and blood urea nitrogen/creatinine 13/0.83 mg/dL. The patient had a positive test result for hepatitis B surface antibody. Tests for hepatitis B surface antigen, anti-hepatitis C virus antibody, and human immunodeficiency virus antigen/antibody were negative. Computed tomography (CT) revealed 3 masses with soft tissue density encasing the transverse and sigmoid colon with a 23 mm-sized abscess in the lower left abdomen. There were pleural masses and fissure nodules in the right lung along the lower portion of the major fissure. Multiple borderline-enlarged lymph nodes were found in the supraclavicular fossa, mediastinum, hilar, and interlobar stations. Enlarged mesenteric and retroperitoneal lymph nodes were also observed (Fig. 1).

**Figure 2.**
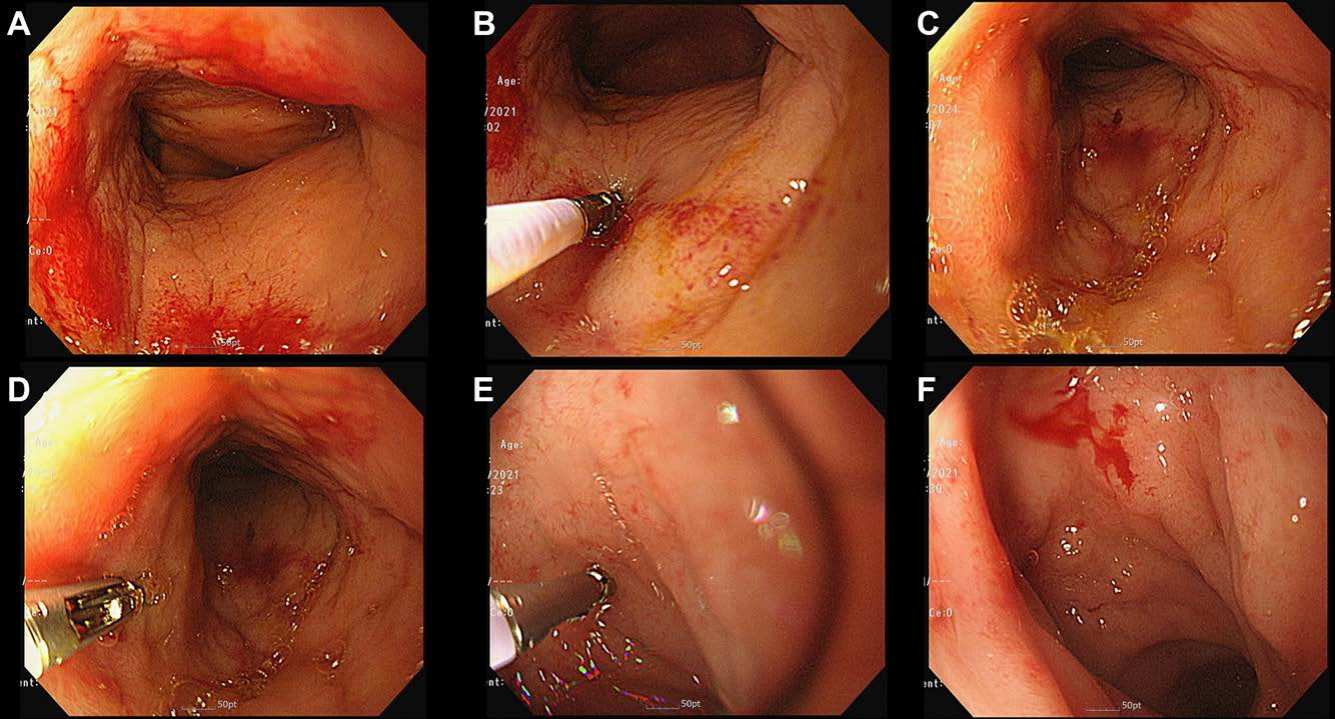
Colonoscopy showing 3 nodular friable mucosal lesions with mild intraluminal stenosis at the sigmoid-descending colon junction (A and B), sigmoid colon (C and D), and rectosigmoid junction (E and F). Biopsy was performed for each lesion.

In the hospital, the patient’s body temperature increased to 39.4 °C. Consequently, we performed percutaneous catheter drainage to drain the intra-abdominal abscess and administered empirical antibiotics. The patient underwent a colonoscopy, which revealed 3 nodular friable mucosal lesions with mild intraluminal stenosis at the sigmoid-descending colon junction, sigmoid colon, and rectosigmoid junction (Fig. 2). Pathological findings from the biopsies revealed chronic colitis with eosinophilic infiltration. The cytology of the percutaneous catheter drainage-drained abscess showed inflammation. Due to the blocking rib, we could not collect a lung mass biopsy. With the impression of eosinophilic colitis, the patient was treated with 30 mg of prednisolone (0.5 mg/kg) for 5 weeks. However, a follow-up CT scan showed no interval change compared to the first one.

**Figure 3.**
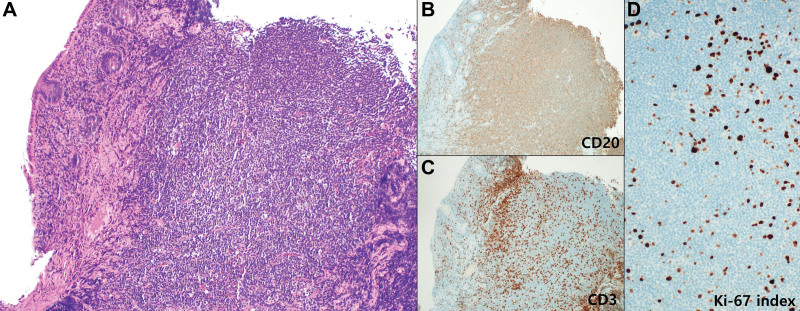
Microscopic, neoplastic small-sized lymphoid cells diffusely infiltrating and replacing the colonic mucosa and submucosa. Several crypts appear destroyed. (hematoxylin and eosin staining, ×100) (A). Immunohistochemical stain showing neoplastic lymphoid cells that are diffusely positive for CD20 (B). CD3 shows reactive T-cells (C), and Ki-67 index is 7.6% (D).

The patient underwent a colonoscopy again. The colonoscopic findings were the same as those in the previous examination. Tissue acquisition with forceps biopsies was performed again, and the pathological findings showed neoplastic small-sized lymphoid cells diffusely infiltrating and replacing the colonic mucosa and submucosa. Several crypts were destroyed. Angulated atypical tumor cells resembled centrocytes and vaguely formed lymphoid follicles. With the impression of lymphoma, additional immunohistochemistry was performed. CD20 was diffusely positive, whereas CD3, CD5, Cyclin D1, and CD10 were negative. The Ki-67 index was 7.6% (Fig. 3). The pathologic findings were consistent with MALT lymphoma, and immunohistochemistry showed that the neoplastic cells were B cell lineage and distinguished from other B cell lymphomas such as diffuse large B cell lymphoma, small lymphocytic lymphoma, follicular lymphoma, and mantle cell lymphoma. Moreover, the immunoglobulin heavy chain (IgH) gene rearrangement test revealed clonal peaks at 319 base pair (tube A, FR1-JH), 250 base pair (tube B, FR2-JH), 105 base pair (tube C, FR3-JH), and 250 base pair (tube D, IGH-D, 6DHs-JH) (see Figure S1, Supplemental Digital Content, http://links.lww.com/MD/K471, which demonstrates clonal peaks in gene rearrangement test of IgH). The IgH gene rearrangement test results meant that there were uncontrolled monoclonal expansions of the specific B cell. Finally, the patient was diagnosed with extranodal marginal zone B-cell lymphoma of the MALT arising in the colon.

Further evaluation with positron emission tomography-CT was performed to determine the extent of the disease. Examination showed colonic MALT lymphoma involving the transverse colon, sigmoid colon, small bowel, right lower lung, and multiple lymph nodes in the abdomen and mediastinum. Based on the Lugano classification system, the lymphoma was diagnosed at stage IV. The patient started to receive chemotherapy with a combination regimen of cyclophosphamide, vincristine, and prednisolone. The first follow-up study after 3 months showed stable disease status without any progression based on response evaluation criteria in solid tumors.

## 3. Discussion

The revised 2016 World Health Organization classification described MALT lymphoma as primarily composed of monocytoid B cells, centrocyte-like cells, and small lymphocytes. Additionally, plasmacytoid differentiation, scattered immunoblasts, and transformed centroblast-like cells may be observed.^[4]^ Colonic MALT lymphoma is an uncommon type of cancer that makes up only 2.5% of all MALT lymphomas.^[6]^ The median age of diagnosis is between 50 to 75 years, and the sex disparity is equal or predominantly female.^[7]^ The pathogenesis of colonic MALT lymphoma remains unclear. Although approximately 20% of colorectal MALT lymphoma cases are *H pylori* positive, there is no evidence of the contribution of *H pylori* to the pathogenesis; moreover, no other chronic inflammation has been proven to be associated with the disease.^[8]^ Within the colorectal tissue, the cecum and ascending colon are the most involved sites, and more than 70% of cases are proximal to the hepatic flexure.^[9]^ Colonic MALT lymphomas are typically asymptomatic.^[10]^ However, it can present with nonspecific gastrointestinal symptoms such as abdominal pain, weight loss, diarrhea, constipation, mucoid stool, hematochezia, and sometimes obstruction.^[11,12]^ On the colonoscopy, single lesion or multinodular polypoid lesions are most common, followed by a subepithelial tumor, flat elevation, mucosal edema, erythema, and loss of vascularity.^[13,14]^ Previously, Jeon et al^[15]^ classified colorectal MALT lymphomas into 4 endoscopic subtypes based on retrospective clinical data: polyposis, epithelial mass, ileitis, and subepithelial tumor, which is the most common endoscopic type.

The patient in this study had 3 nodular friable mucosal lesions at the sigmoid-descending colon junction, sigmoid colon, rectosigmoid junction, and an intra-abdominal abscess (Fig. 2). Initial colonoscopic biopsy revealed chronic colitis with eosinophilic infiltration, which corresponded to eosinophilic colitis. However, the endoscopic findings were nonspecific, and the patient had other lesions of the lung and lymph nodes, which warranted further evaluation. Additionally, steroid therapy for eosinophilic colitis was not as effective as expected. Although the initial biopsy did not show any evidence of malignancy, the patient had a colonic mass with enlarged intra-abdominal and intrathoracic lymph nodes, implying the possibility of malignancy. Therefore, we performed a repeat biopsy that revealed a final pathologic report of MALT lymphoma of the colon.

Clinical suspicion based on endoscopic findings is the first and most important step to differentiate the diagnosis, especially from malignancy. In previous data, however, the first endoscopic impression of lymphoma was made in only 18% of confirmed colorectal MALT lymphoma patients.^[15]^ Moreover, although tissue acquisition and investigation of a microscopic pathological finding are key steps in making a diagnosis, given the chance of an incomplete biopsy, a single biopsy cannot guarantee the representativity of the lesion. Hence, a repeat biopsy is necessary to make a definite diagnosis when there is a discoordination between the clinical features and pathological findings. Many reports have highlighted the importance of repeat biopsy during malignancy diagnosis, especially in cancers that do not have pathognomonic immunohistochemistry.^[16–18]^ Repeat biopsy should be considered whenever the diagnosis is inconclusive or the patient has an atypical clinical course.

MZL staging usually adopts the Ann Arbor staging system. However, gastrointestinal MZL is staged according to the modified Ann Arbor scheme, the Lugano staging system. This modified system incorporates indices corresponding to the depth of mucosal invasion and proximity of the affected lymph nodes to the primary lesion.^[19]^ Unlike most other indolent lymphomas, extranodal MZL frequently presents at a localized stage, wherein 70% of cases are stage I or II, and the risk of systemic dissemination is low.^[20]^ Stage IV MALT lymphomas have seldom been reported. Most data reporting colonic MALT lymphomas have shown a small proportion of patients with stage IV disease.^[4,15]^ The patient in this case presented with disseminated lymph node metastasis, lung involvement, and an intra-abdominal abscess that required drainage. According to the Lugano modification of the Ann Arbor system, the patient had stage IV disease.

This study is a single case report and has a limitation in defining the specific features of stage IV colonic MALT lymphoma. However, the study shows the importance of clinical suspicion and repeat biopsy in patients with atypical colon lesions and clinical features to diagnose colonic MALT lymphoma, which is very rare and diagnostically challenging.

To the best of our knowledge, this is the first report of advanced-stage colonic MALT lymphoma with an abscess mimicking eosinophilic colitis.

## Author contributions

**Conceptualization:** Kangkook Lee, Ju Yup Lee.

**Data curation:** Kangkook Lee, Jin Wook Lee, Hye Ra Jung.

**Methodology:** Hye Ra Jung, Myeongsoon Park, Ju Yup Lee.

**Resources:** Jin Wook Lee, Myeongsoon Park.

**Supervision:** Kwang Bum Cho, Ju Yup Lee.

**Writing – original draft:** Kangkook Lee.

**Writing – review & editing:** Hye Ra Jung, Kwang Bum Cho, Ju Yup Lee.

## Supplementary Material


