# Supplementary material for: A case of colonic MALT lymphoma with intra-abdominal abscess and lung metastasis: A case report
Source: Medicine (Baltimore). 2023 Oct 27;102(43):e35778. doi: 10.1097/MD.0000000000035778 (PMC10615456; doi:10.1097/MD.0000000000035778)
Supplement: Supplementary file 1 [file medi-102-e35778-s001.pdf]

Applied Biosystems
GeneMapper Software 5

20220307_IVS

Sample File	Sample Name	Panel	SQI	SQ
77A-A07-2022-03-04-16-09-17-07.fsa	77A	IGH-A		

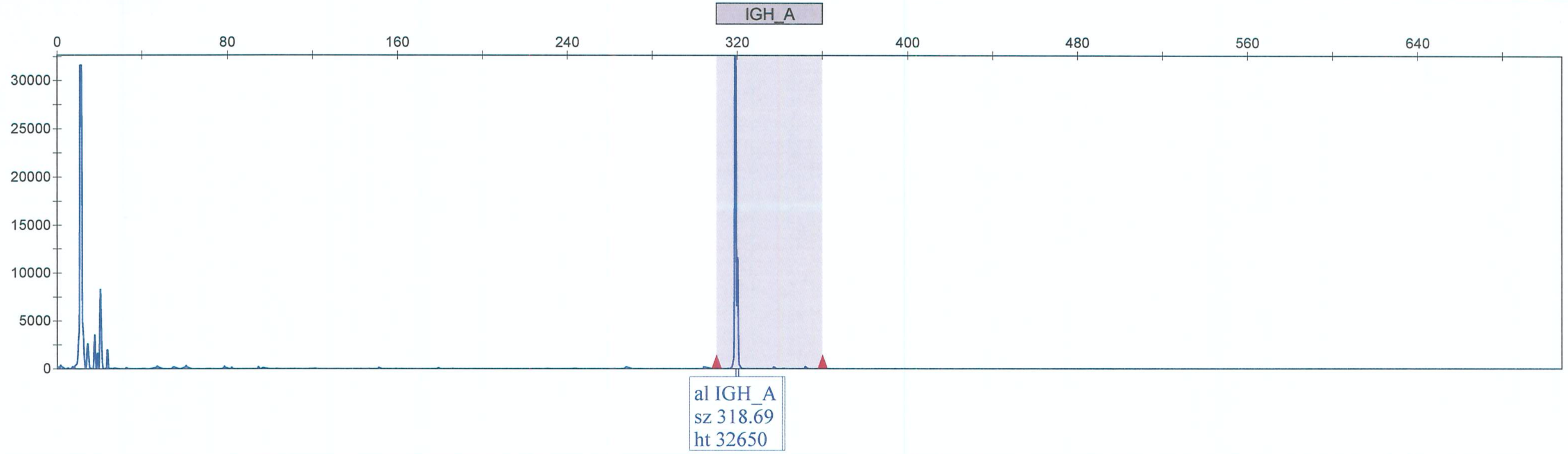

77A-A07-2022-03-04-16-09-17-07.fsa	77A	IGH-A		

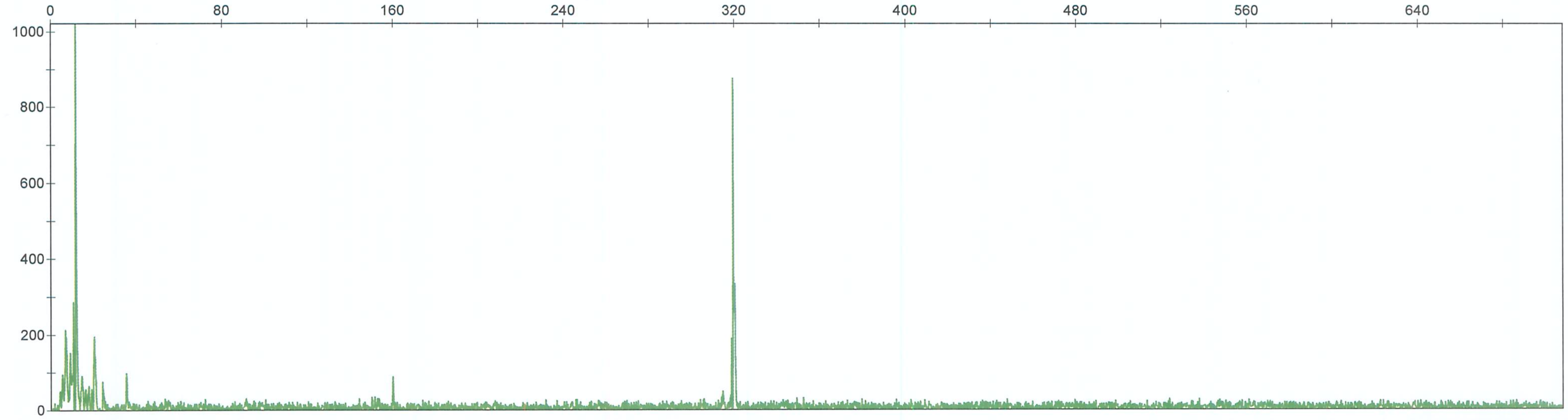

Applied Biosystems
GeneMapper Software 5

20220307_IVS

Sample File	Sample Name	Panel	SQI	SQ
77B-B07-2022-03-04-16-09-17-07.fsa	77B	IGH-B		

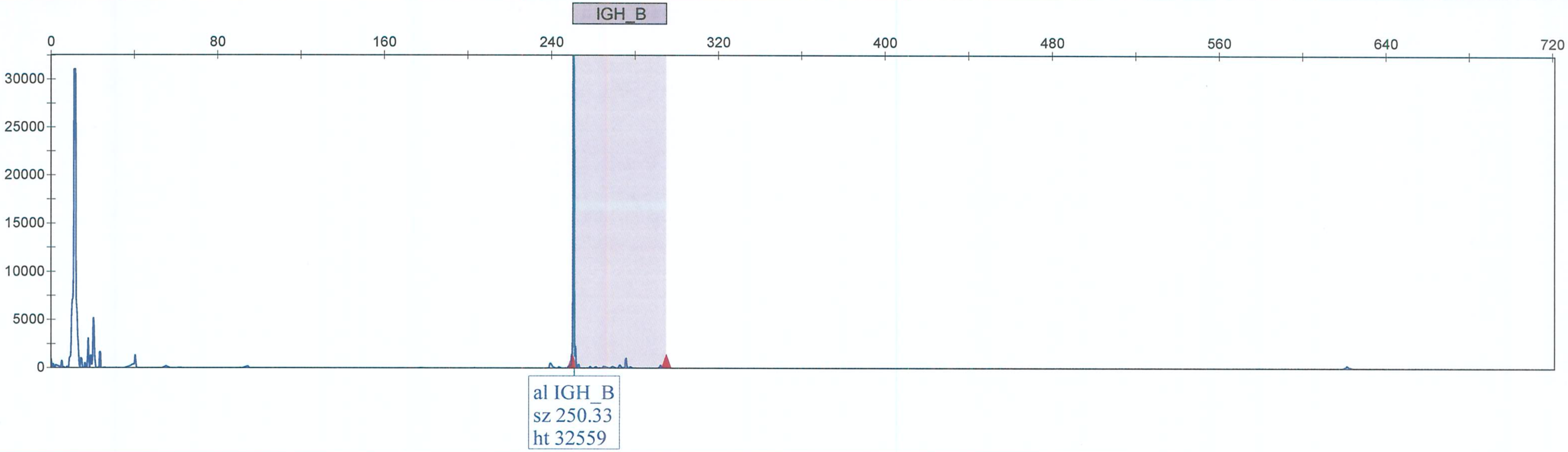

77B-B07-2022-03-04-16-09-17-07.fsa	77B	IGH-B		

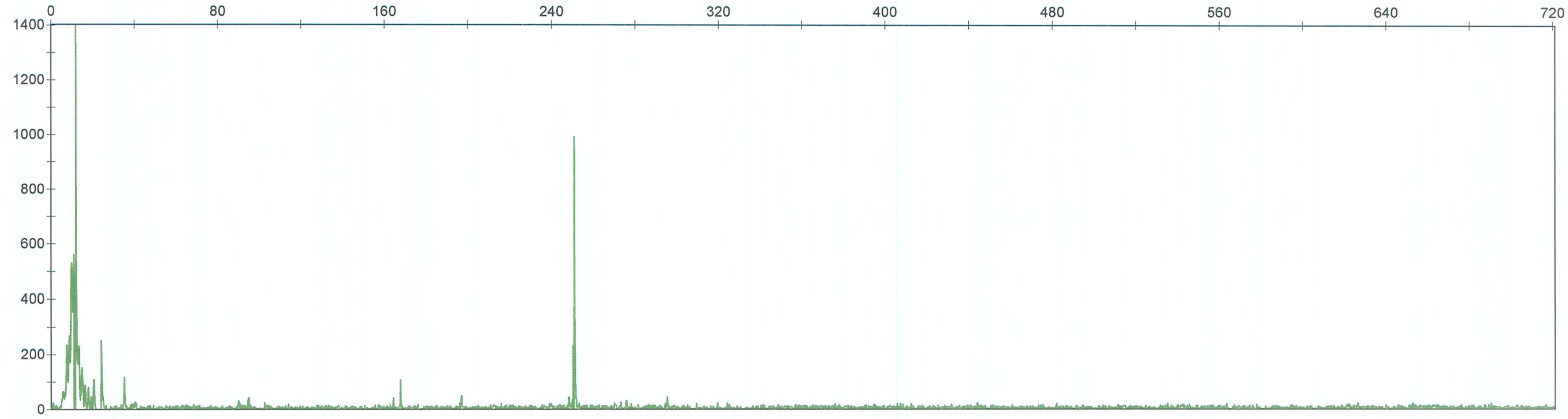

Applied Biosystems
GeneMapper Software 5

20220307_IVS

Sample File	Sample Name	Panel	SQI	SQ
77C-C07-2022-03-04-16-09-17-07.fsa	77C	IGH-C		

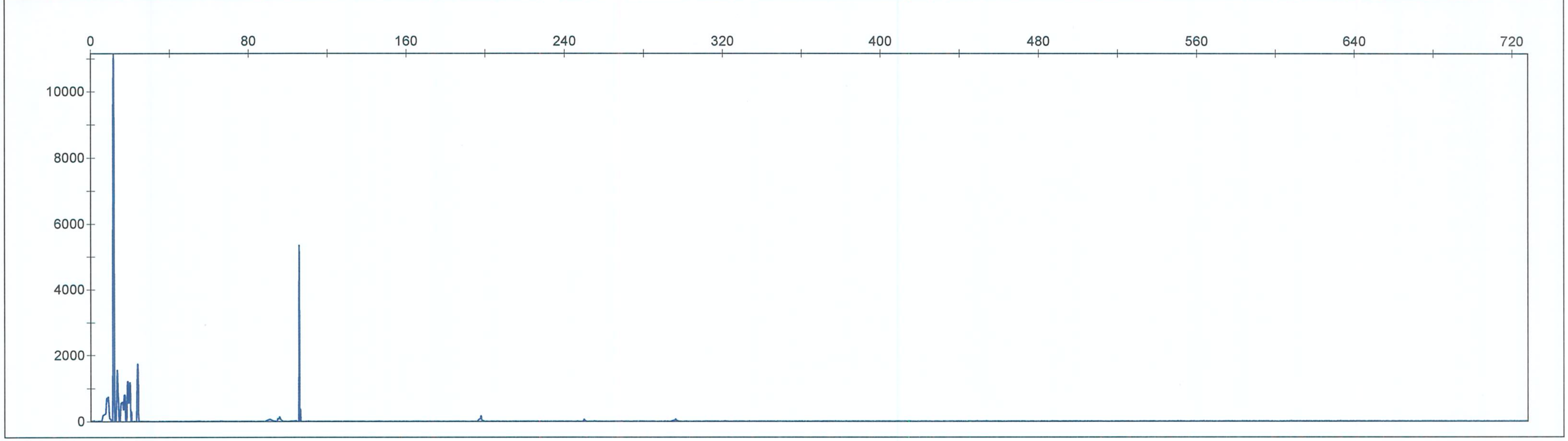

77C-C07-2022-03-04-16-09-17-07.fsa	77C	IGH-C		

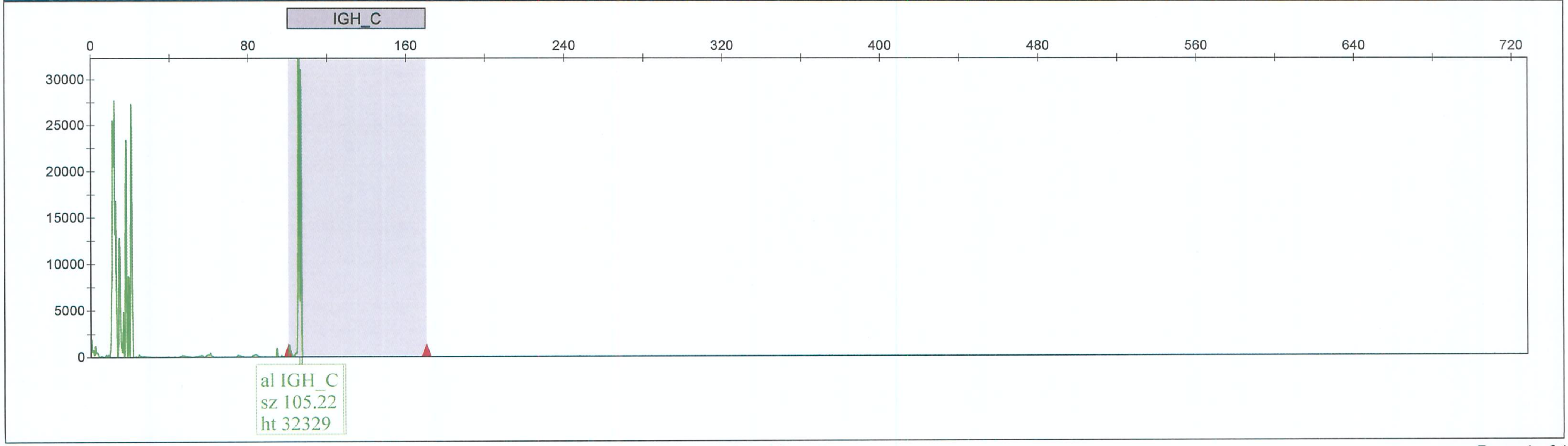

20220307_IVS

Sample File	Sample Name	Panel	SQI	SQ
77D-D07-2022-03-04-16-09-17-07.fsa	77D	IGH-D		

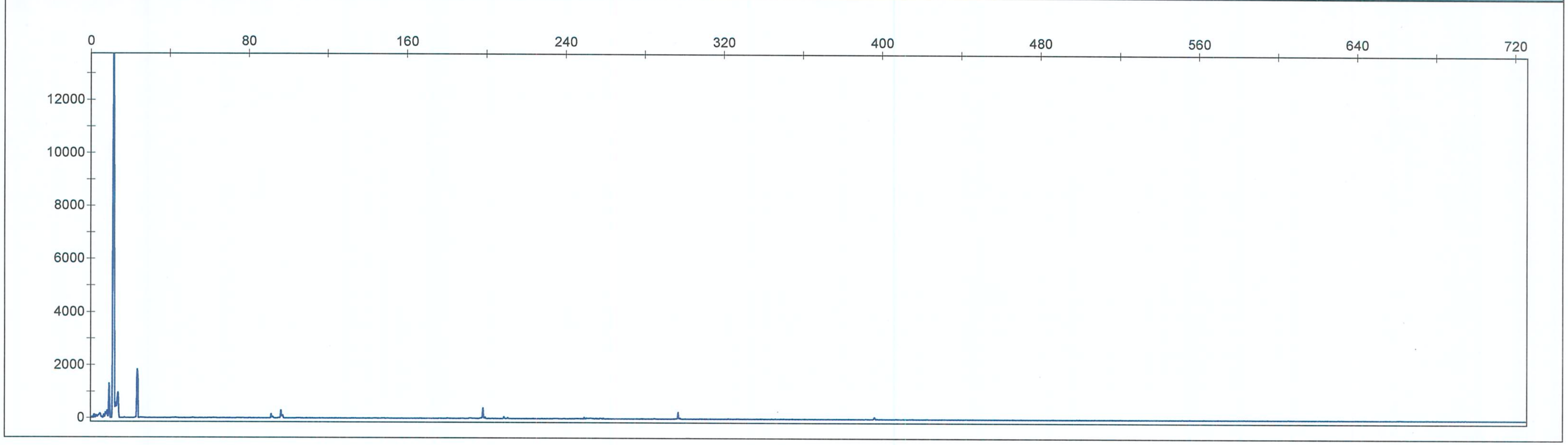

77D-D07-2022-03-04-16-09-17-07.fsa	77D	IGH-D		

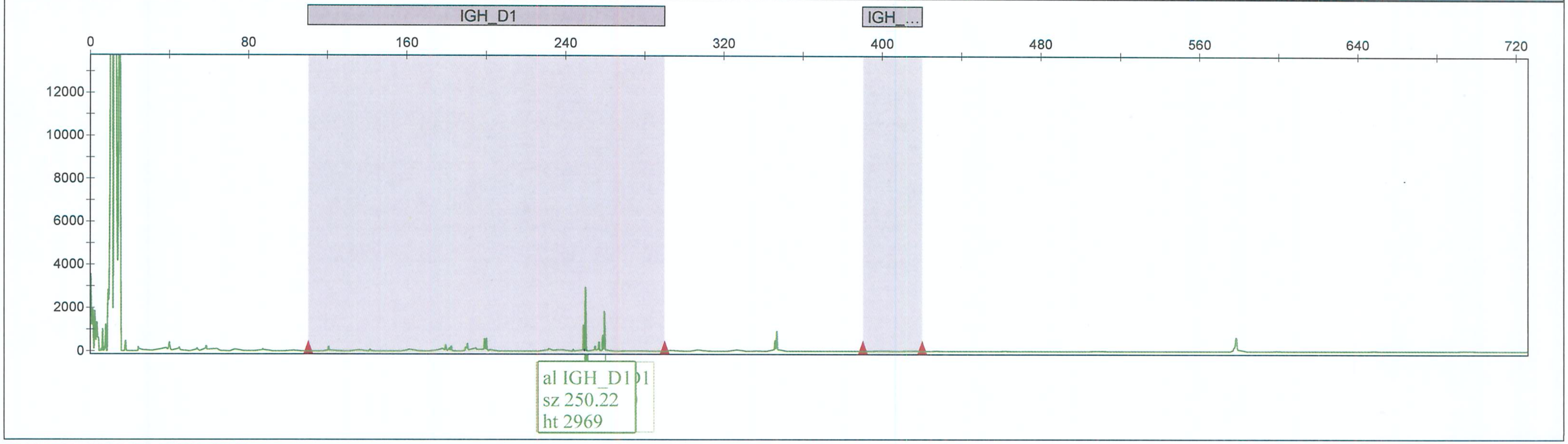

Applied Biosystems
GeneMapper Software 5

20220307_IVS

Sample File	Sample Name	Panel	SQI	SQ
77E-E07-2022-03-04-16-09-17-07.fsa	77E	IGH-E		

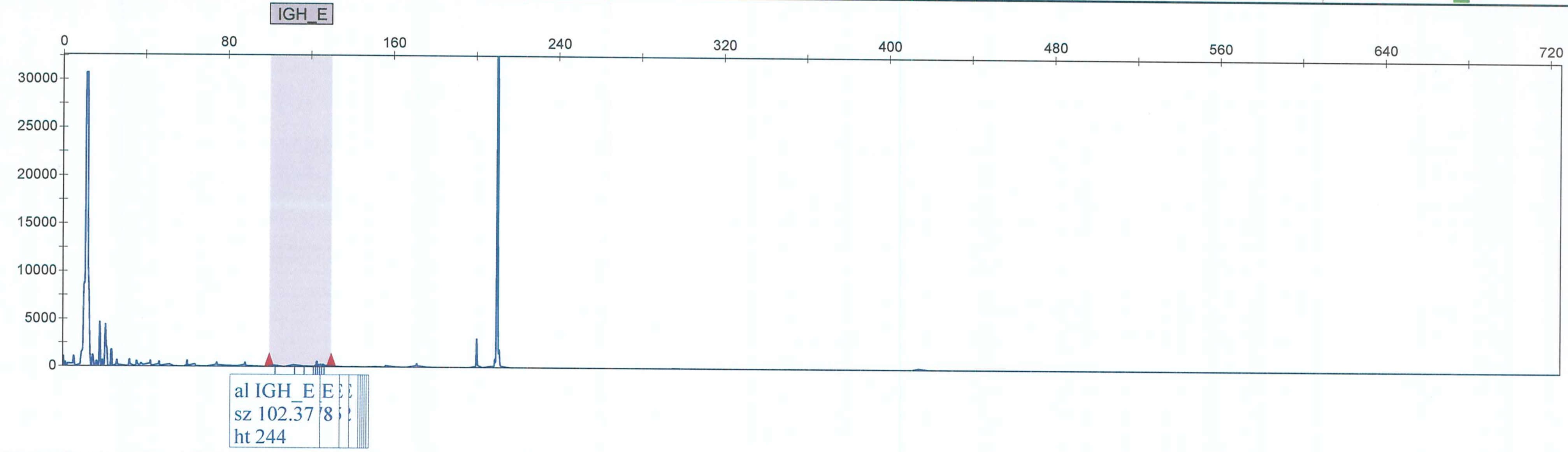

77E-E07-2022-03-04-16-09-17-07.fsa	77E	IGH-E		

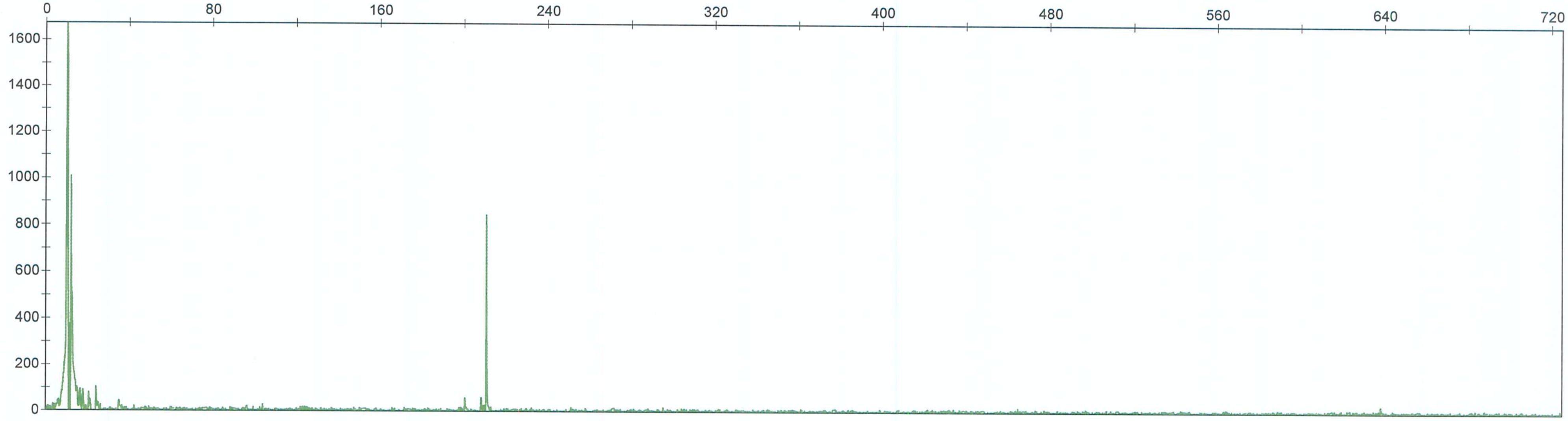

Applied Biosystems
GeneMapper Software 5

20220307_IVS

Sample File	Sample Name	Panel	SQI	SQ
77S-F07-2022-03-04-16-09-17-07.fsa	77S	None		

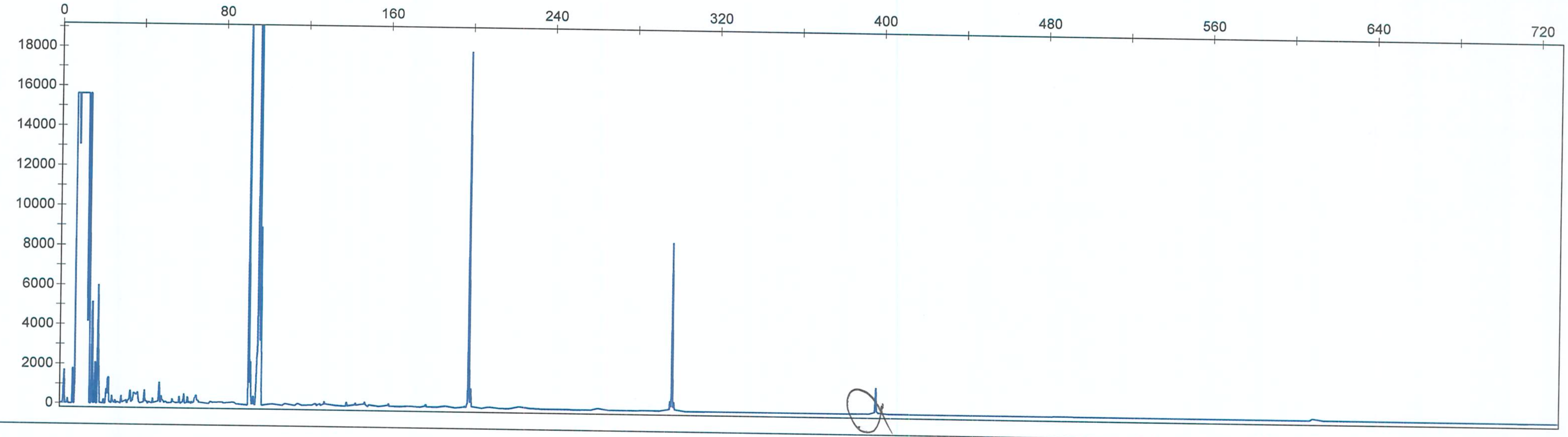

77S-F07-2022-03-04-16-09-17-07.fsa	77S	None		

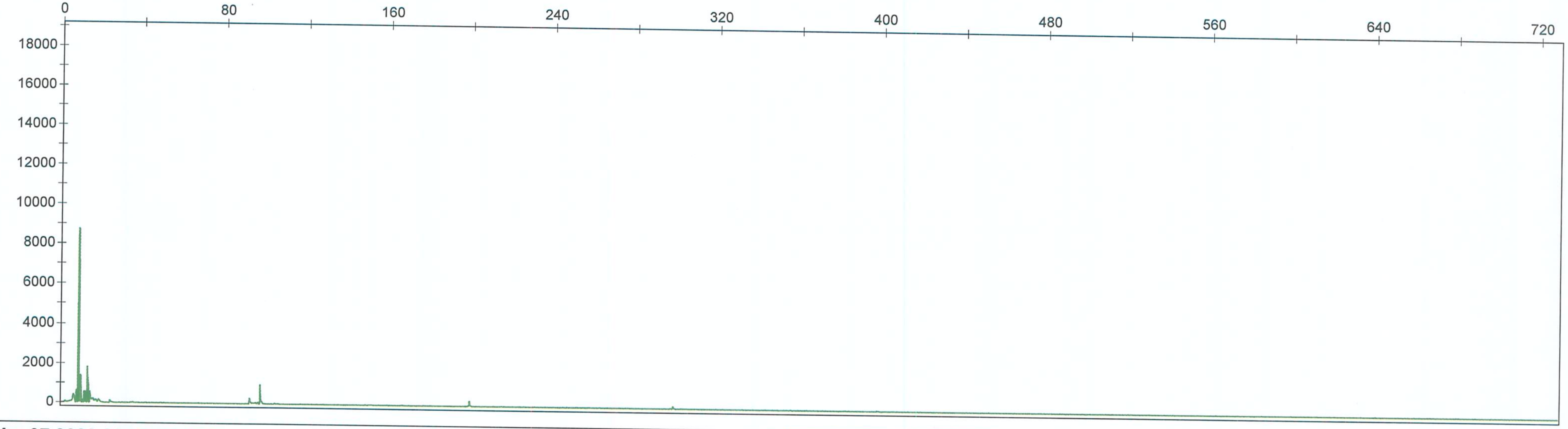

Mon Mar 07,2022 08:13AM, PST

Printed by: gm
